# Room temperature magneto-optic effect in silicon light-emitting diodes

**DOI:** 10.1038/s41467-017-02804-6

**Published:** 2018-01-26

**Authors:** F. Chiodi, S. L. Bayliss, L. Barast, D. Débarre, H. Bouchiat, R. H. Friend, A. D. Chepelianskii

**Affiliations:** 1 Centre de Nanosciences et de Nanotechnologies, CNRS, Univ. Paris-Sud, Université Paris-Saclay, C2N-Orsay, Orsay, 91405 France; 2 Laboratoire de Physique des solides, CNRS, Univ. Paris-Sud, Université Paris-Saclay, LPS-Orsay, Orsay, 91405 France; 3 Cavendish Laboratory, University of Cambridge, J. J. Thomson Avenue, Cambridge, CB3 OHE UK

## Abstract

In weakly spin–orbit coupled materials, the spin-selective nature of recombination can give rise to large magnetic-field effects, e.g. on the electro-luminescence of molecular semiconductors. Although silicon has weak spin–orbit coupling, observing spin-dependent recombination through magneto-electroluminescence is challenging: silicon’s indirect band-gap causes an inefficient emission and it is difficult to separate spin-dependent phenomena from classical magneto-resistance effects. Here we overcome these challenges and measure magneto-electroluminescence in silicon light-emitting diodes fabricated via gas immersion laser doping. These devices allow us to achieve efficient emission while retaining a well-defined geometry, thus suppressing classical magnetoresistance effects to a few percent. We find that electroluminescence can be enhanced by up to 300% near room temperature in a seven Tesla magnetic field, showing that the control of the spin degree of freedom can have a strong impact on the efficiency of silicon LEDs.

## Abstract (web summary)

Silicon is an important material in spintronics but its inefficiency in light emission limits the optical probes for spin transport. Here Chiodi et al. develop ultra-doped silicon light-emitting devices and show that electroluminescence can be used to probe spin phenomena in silicon even at room temperature.

## Introduction

Spintronic effects in systems with weak spin–orbit coupling have attracted considerable attention due to their rich fundamental physics and potential for device applications^1–4^. A class of these effects can be measured optically^5–7^, providing direct insight into phenomena such as spin-dependent recombination, where only the singlet state of an electron-hole pair can recombine radiatively back to the ground state. As external magnetic fields can change the spin statistics and energy levels in the sample, magneto-electroluminescence (MEL) effects have been seen as the hallmark of spin-dependent recombination phenomena and have given important insight into the role of spin in organic materials used for light-emitting diodes (LEDs)^8–10^. These spintronic effects can then be harnesseed to provide very senstive magnetic field sensors, sensible to external magentic fields of only a few mTesla comparable with the fluctuating hyperfine fields inside organic materials^11–13^ or to engineer new light-emitting device architectures through reverse intersystem crossing^14^.

Similar to molecular semiconductors, silicon has weak spin–orbit coupling, but emission is much less efficient due to silicon’s indirect bandgap, making analogous magneto-optic studies challenging and requiring careful engineering to prepare efficient LEDs^15–17^. In addition, observing spin-dependent MEL in silicon requires that the magnetic field and device currents are parallel to effectively suppress classical magnetoresistance (MR) contributions, which can enhance MR in silicon up to spectacular values even at room temperature^18–22^.

Here we address both of these challenges by developing a new fabrication method for efficient silicon LEDs (SiLEDs) using an original doping technique, gas immersion laser doping (GILD), and investigate spin-dependent recombination in SiLEDs. The GILD process^23–26^ allows us to reach doping levels well beyond the solubility threshold, which, as we describe below, gives rise to efficient emission, while retaining the well-defined planar geometry necessary to align electric and magnetic fields. Using our SiLEDs, we find that when classical MR effects are suppressed, EL can be substantially enhanced under a magnetic field near room temperature. We explain this phenomenon using a model of spin-dependent recombination^27–29^ of electron-hole pairs and use our analysis to estimate the exchange energy of weakly bound excitons in silicon. Our experiments provide an optoelectronic approach to probe the spin statistics of carriers in silicon—a material which is an excellent candidate for scalable spin quantum computing^30–32^. They also highlight the importance of controlling the spin degree of freedom for the efficiency of silicon light-emitting devices.

## Results

### Description of the system

**Fig. 1:**
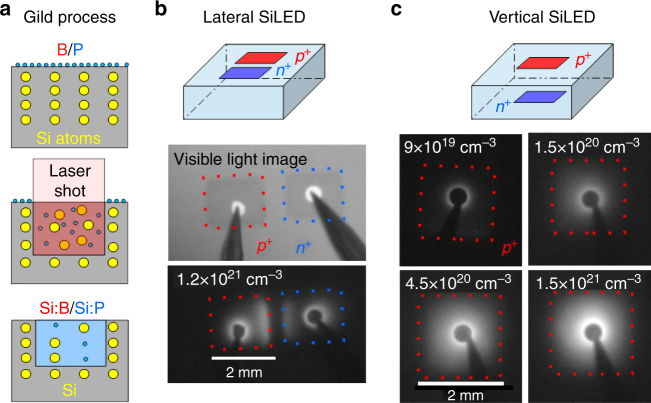
Silicon light-emitting diodes from gas immersion laser doping (GILD). **a** Schematics of the GILD doping process: chemisorbtion of the dopant gas (PCl_3_/BCL_3_); laser melting of the bulk Si and dopant incorporation in the liquid phase; Si:P or Si:B crystal epitaxy during solidification. **b** Schematic of lateral devices and infrared images of a silicon light-emitting device (SiLEDs) (1.2 × 10^21^cm^−3^) biased at 20 mA and at room temperature. **c** Schematic of vertical devices and infrared images of SiLEDs biased at 20 mA at room temperature for different doping levels (9 × 10^19^, 1.5 × 10^20^, 4.5 × 10^20^, 1.5 × 10^21^ cm^−3^)

We start by describing the fabrication procedure of the GILD SiLEDs (Fig. 1a) and the physical mechanism behind their enhanced efficiency, before discussing the MEL response of these devices. The Si LEDs were prepared by doping two 2 × 2 mm^2^ spots with opposite polarities *p*+/ *n*+ on a n-Si [100] substrate of resistivity 45 Ω cm and thickness 700 μm using the GILD technique (Fig. 1a). A precursor gas PCl_3_ (BCl_3_) for *n*+ (*p*+) doping is injected into an ultra-high vacuum chamber, where it saturates the chemisorbtion sites on the Si surface. The silicon is melted up to the desired doping depth by a XeCl 308 nm excimer laser of 25 ns pulse duration and tunable energy. This induces the rapid diffusion of the chemisorbed B into the liquid Si region (Fig. 1a). As a result, a box-like Si:P/Si:B crystal is grown by liquid phase epitaxy on the underlying Si substrate^25^. Multiple chemisorbtion/melting cycles determine the dose of active dopants introduced in the doped layer. Due to the high recrystallization velocity (4 m s^−1^), the Si undergoes quenching, and high dopant concentrations beyond the solubility limit (10^20^ cm^−3^ for Si:B)^33^ can be reached without introducing defects. In our experiments, we varied the doping concentration in the range 4.5 × 10^19^ to 5 × 10^21^cm^−3^, while keeping the doping depth constant at 80 nm (pulse energy 960 mJ cm^−2^). Ti(15 nm)/Al(200 nm) electrodes were deposited on top of the doped spots after buffered hydrofluoric acid (BHF) deoxidation.

### Device electroluminescence

We investigated EL from GILD SiLEDs in two different device geometries where the *p*+ and *n*+ spots were placed laterally, without overlap, on the same Si surface (Fig. 1b) and in a vertical geometry where the *n*+/ *p*+ were prepared on top of each other on opposite sides of the Si wafer (Fig. 1c). The alignment between top and bottom spots in the vertical geometry was achieved by looking through the silicon wafer with an infrared camera, which allowed to see the bottom GILD spots. This camera also allowed to record characterization images of EL devices. Images in the lateral geometry (Fig. 1b) show that EL occurs mainly at the heavily doped GILD spots on both *n* + and *p* + sides, whereas the undoped region in between the two spots remains dark. This shows that EL is enhanced near the *n* + and *p *+ interfaces as compared with bulk Si. This observation is supported by the strong improvement of the device brightness as the GILD doping concentration increases (see Fig. 1c). The external quantum efficiency (EQE) for our brightest devices is around 0.05%, which is comparable with the highest reported values for devices without anti-reflection treatment^15^.

**Fig. 2:**
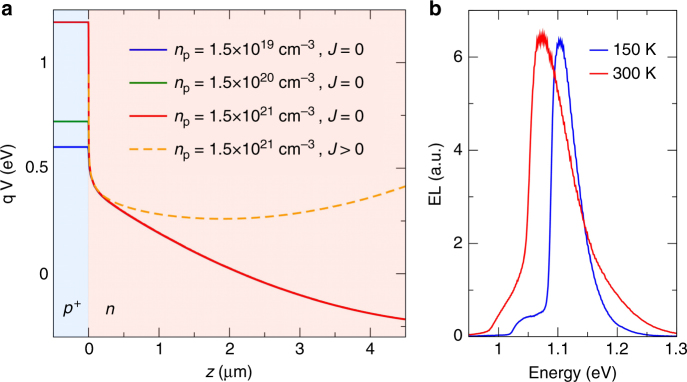
Origin of enhanced emission in gas immersion laser doping silicon light-emitting devices. **a** Simulated electrostatic potential at the *p* + / *n* interface as a function of the vertical distance *z* to the *p* + / *n* interface. Full curves show different *p* + doping levels without external bias. The dashed curve shows the formation of a potential minimum under forward bias at a current density of *J*=2 × 10^4^ Am^−2^ for the highest doping level. **b** Emission spectra from a lateral device with 1.2 × 10^21^cm^−3^ doping

The physical origin of the EL enhancement can be understood from the electrostatic profile within the devices, which we model using drift-diffusion simulations of the *p* +/*n* interface accounting for the Fermi statistics in the highly doped regions^34^. The simulated distributions of the electrostatic potential *V*(*z*) are shown on Fig. 2a. In unbiased devices (see Supplementary Figure 1 for a more detailed discussion), the potential *V*(*z*) inside the *n* region (*z* > 0) is almost independent on the *p *+ doping concentration *n*_p_; however, a steep potential step forms at the *p* +/ *n* interface, its height increasing with doping, creating a barrier that electrons have to overcome to leave the device. For parameters corresponding to the brightest devices the barrier is near an eV high and thermally activated transport is effectively prohibited. The *p* + region thus has the role of an electron blocking layer, whereas the *n *+ region will similarly act as a hole blocking layer. Such layers are known to enhance the efficiency of organic LEDS^35,36^. When devices are biased the potential *V*(*z*) remains constant in the *p *+ region and near the *p* +/*n* interface as the applied potential will mainly drop across the intrinsic weakly doped regions, which have much larger resistivity than the highly doped *p* + region. A potential minimum therefore appears at sufficiently high forward bias near the *p *+/*n* and *n*/*n *+ interfaces. In these regions, located at a vertical distance of 1–2 μm away from the interfaces, the internal electric field vanishes favoring radiative recombination, as a built-in electric field would otherwise drive electron-hole pairs apart. Similarly, the minority carriers (holes) predominantly recombine at the *n* + interface as can be seen from the weaker EL observed from the *n* + spot in lateral devices (see Fig. 1b). Finally, EL spectra in Fig. 2b (see also Supplementary Figure 2) are in very good agreement with previously reported lineshapes for bulk Si^37–39^ in agreement with our model predicting emission in the weakly doped Silicon a micrometer away from the *p* +/*n* and *n* +/*n* interfaces.

### SiLED magneto-electroluminescence

Having described the structure of our devices and the physical mechanism behind their enhanced EL efficiency, we now use them to study the dependence of the EL on magnetic field in a vertical SiLED device (doping 3 × 10^21^ cm^−3^); similar data were obtained on a device with 1.5 × 10^21^ cm^−3^ doping. For this experiment, devices where mounted inside an optical access magnet (Oxford Instruments) and the EL was collected by a Ge photo-detector outside the cryostat. The SiLEDs were DC biased and the input of the Ge photodetector was chopped at 230 Hz to enhance sensitivity.

**Fig. 3:**
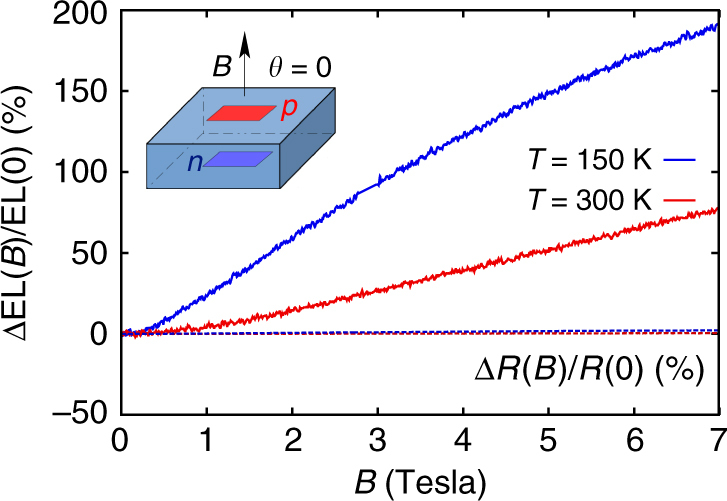
Magneto-electroluminescence in silicon light-emitting devices. Comparison between MEL and MR effects in a vertical SiLED under a perpendicular magnetic field (magnetic field tilt angle *θ* = 0 relative to current lines) at 300 and 150 K, where ΔEL(*B*) = EL(*B*)−EL(0) and Δ*R*(*B*) = *R*(*B*) − *R*(0). The DC forward bias current was 10 mA

To study the role of spin in the MEL, we first applied a magnetic field *B* perpendicular to the device surface, i.e., parallel to the internal electric field. We obtained a vanishing classical MR as shown in Fig. 3, where we observe only a weak residual MR in the 1% range both at 300 and 150 K. The accuracy of this MR cancellation can seem surprising given the lack of electrical insulation around the spots. However, close to the onset voltage of the diode, the voltage drop mainly occurs accross the few micron wide depletion region between the *p* + and *n* regions (the extent of the depletion region is shown on Supplementary Figure 1) and the current lines cannot bend significantly considering the large spot size 2 mm × 2 mm. Figure 3 also plots the MEL response, which shows a drastically different behaviour. Compared with the MR signal, the EL exhibits a two orders of magnitude stronger dependence on the magnetic field, with ΔEL(*B*)/EL(0) = 75% at room temperature and an even higher ΔEL(*B*)/EL(0) = 290% value at 150 K (for a 5 mA current). The striking difference in magnitude of the MEL signal over the MR suggests that the magnetic field is increasing radiative recombination. As the EL quantum efficiency is low in silicon, a strong change in EL has little effect on the total current and hence the MR.

**Fig. 4:**
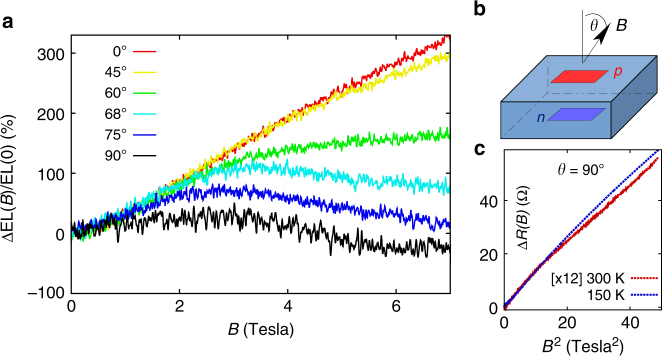
Angular dependence of magneto-electroluminescence in silicon light-emitting devices. Evolution of the magneto-electroluminescence as a function of the tilt angle *θ* between the magnetic field and the 5 mA DC current at 150 K (data shown in **a**, experiment geometry is sketched in **b**). **c** MR response measured at *θ *= 90°, which displays the classical *B*^2^ dependence that contrasts with the MEL field dependence

To further investigate the origin of the strong MEL effect in SiLEDs, we measured the MEL response as a function of the angle of the magnetic field relative to current lines Fig. 4. For moderate tilt angles *θ* below 45°, the MEL signal is nearly independent on the tilt. At larger tilt angles *θ* below 75°, the MEL is unchanged at low-magnetic fields but decreases from the low tilt angle behaviour at high magnetic fields. We attribute this decrease to the magneto-diode effect^40–42^: the in-plane component of the magnetic field bends electron and hole trajectories so that they cross a larger distance through the device and thus have a higher non radiative-recombination probability. A significant bending of current lines can occur in perpendicular electric and magnetic fields when the ratio *μ*_xy_/*μ*_xx_ between Hall and longitudinal mobility is large. This quantity is around 1 at room temperature for *B *= 7 T and around 5 at 150 K (using the mobility values for a 45 Ω cm electron doped Si: *μ*_xx_ = 1.4 × 10^3^ cm^2^ V^−1^ s^−1^ at room temperature and *μ*_xx_ = 7 × 10^3^ cm^2^ V^−1^ s^−1^ at 150 K). The negative magneto-diode contribution to MEL is further enhanced in lateral devices with longer current paths through the sample (see Supplementary Figure 4), with a negative MEL amplitude comparable with the ratio *μ*_xy_/*μ*_xx_.

The full MEL response is thus a superposition of a positive MEL component that depends only weakly on the *B*-field direction and a negative magneto-diode effect component that contributes more strongly at large tilt angles. A purely magnetodiode contribution would have the same *B* dependence as the MR, which increases with the parallel magnetic field as *B*^2^, as expected from the Drude law (see Fig. 4), in contrast with the observed MEL signal that displays a linear dependence with *B*, except at the lowest fields. As the angle and magnetic field dependence of the positive MEL effect are very different from the MR, we infer that the MEL is not determined by transport scattering times and may be related to spin degrees of freedom.

## Discussion

**Fig. 5:**
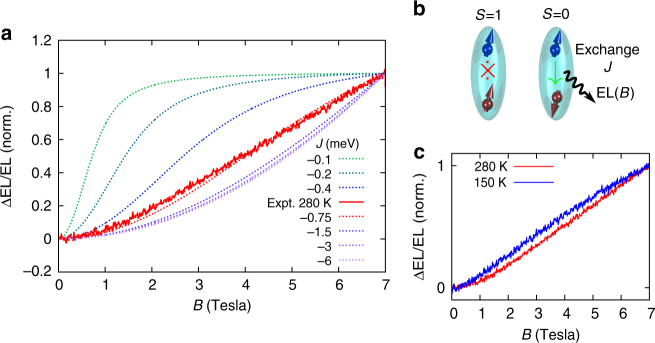
Electron-hole spin-dependent recombination theory for the magneto-electroluminescence. Simulated magneto-electroluminescence using the model described in the text. Normalised simulations are shown in **a** for varying electron-hole exchange energy *J* alongside the experimental data at 300 K (a comparison between normalised 300 and 150 K data is shown in **c**). The characteristic saturation field of the magneto-electroluminescence is determined by the electron-hole exchange energy. Fitting to the 300 K experimental data gives an exchange energy *J *= −0.75 meV. Panel **b** illustrates the singlet and triplet spin pairings of a weakly bound electron/hole pair for which only the singlet state is emissive

The MEL effect observed in SiLEDs when the current and magnetic field are parallel (*θ* = 0) can be explained as arising from the spin-dependent recombination of weakly bound electron-hole pairs within the devices, in analogy with the models developed by Kaplan et al.^27^ and Merrifield^28^. The ability of an electron-hole pair to recombine radiatively is determined by the overlap of the electron-hole pair wavefunction with the spin-zero singlet ground state—radiative recombination will only be efficient for electron-hole pairs in an *S* = 0 singlet configuration, the recombination from an *S* = 1 triplet electron-hole pair being much less efficient (Fig. 5b). As the magnetic field is changed, the electron-hole eigenstates—which are in general not pure singlet or triplet spin states—are modified, altering their singlet overlap. As we explain below, this change in the electron-hole pair wavefunctions gives rise to a change in EL and can explain the lineshapes observed experimentally. Importantly, this effect is independent of the direction of the magnetic field and can therefore describe the angular-dependent MEL observations (Fig. 4). We note that although spin-dependent free-carrier recombination has previously been studied through circularly polarized emission^43–45^, here we invoke spin-dependent recombination of weakly bound exchange coupled electron-hole pairs to explain our MEL effects.

We start by considering the kinetic equation for the population of transient electron-hole pairs that are formed by brief collisions in the device recombination zone near room temperature. The population *X*_n_ of transient pairs with spin-eigenstates $\left| n \right\rangle$ is expected to follow the following rate equations:

1 $$\dot X_{\mathrm{n}} = G_{\mathrm{n}} - \gamma _{\mathrm{s}}\alpha _{\mathrm{n}}X_{\mathrm{n}} - \gamma X_{\mathrm{n}}.$$

Here, *G*_n_ is the electron-hole pair generation rate. The second term describes the probability of (spin-dependent) radiative recombination during collision events, with a rate *γ*_s_. This term is proportional to the overlap of the electron-hole pair wavefunction with the spin-singlet state $\alpha _{\mathrm{n}} = \left| {\left\langle {S|n} \right\rangle } \right|^2$, where $\left| S \right\rangle$ is the singlet state. The final (spin-independent) *γ* term reflects both the probability of escape from the shallow potential well where radiative recombination occurs in our devices and the rate of nonradiative relaxation. Solving this in steady-state for the total emission from the electon-hole pairs ${\mathrm{EL}} = \mathop {\sum}\nolimits_{\mathrm{n}} \gamma _{\mathrm{s}}\alpha _{\mathrm{n}}X_{\mathrm{n}}$ we find

2 $${\mathrm{EL}} \propto \mathop {\sum}\limits_{\mathrm{n}} \frac{{G_{\mathrm{n}}\alpha _{\mathrm{n}}}}{{1 + \varepsilon \alpha _{\mathrm{n}}}},$$

where *ε* = *γ*_s_/*γ*. This sum depends non-linearly on the singlet projections {*α*_n_} and so a magnetic-field induced change in these can give rise to a change in emission. To compute EL from Equation 2, we calculate the singlet projections by diagonalizing the following electron-hole pair spin Hamiltonian

3 $$\hat H = \underbrace {g_{\mathrm{e}}\mu _{\mathrm{B}}{\mathbf{B}} \cdot {\hat{\mathbf S}}_{\mathrm{e}}}_{\mathrm{electron}} + \underbrace {g_{\mathrm{h}}\mu _{\mathrm{B}}{\mathbf{B}} \cdot {\hat{\mathbf S}}_{\mathrm{h}} + \lambda {\hat{\mathbf L}}_{\mathrm{h}} \cdot {\hat{\mathbf S}}_{\mathrm{h}}}_{\mathrm{hole}} + \underbrace {J{\hat{\mathbf S}}_{\mathrm{e}} \cdot {\hat{\mathbf S}}_{\mathrm{h}}}_{\mathrm{exchange}},$$

where *g*_e_, *g*_h_ are electron and hole *g*-factors, *μ*_B_ the Bohr magneton, **B** the applied field, ${\hat{\mathbf S}}_{\mathrm{e}},{\hat{\mathbf S}}_{\mathrm{h}}$ are the electron and hole spins, *λ* is the spin–orbit parameter for the hole with ${\hat{\mathbf L}}_{\mathrm{h}}$ the hole orbital angular momentum, and *J* is the electron-hole exchange coupling. We note that the form of the effective spin Hamiltonians are similar for the transient interacting electron hole pairs considered here and tightly bound excitons, the main difference being the amplitude of the exchange interaction *J* compared with temperature with $J \ll k_{\mathrm{B}}T$ for transient electon-hole pairs as opposed to bound excitons.

Figure 5 shows the change in EL as a function of magnetic field calculated from Equation 2 for various values of the electron-hole exchange parameter *J*, using *λ* = −44 meV^46,47^, *g*_e_ = *g*_h_ = 2^48,49,50^, Boltzmann populations, i.e., $G_{\mathrm{n}} \propto {\mathrm{e}}^{ - E_{\mathrm{n}}/k_{\mathrm{B}}T}$ where *E*_n_ is the electron-hole pair energy, *k*_B_ is Boltzmann’s constant, and *T* = 300 K is the temperature. (This population distribution assumes that electron-hole pairs thermalize within their encounter time, but due to the high temperatures involved, similar results are obtained in the fully unpolarized limit where the generation rates are equal for each spin sublevel.) We note that for $\varepsilon = \gamma _{\mathrm{s}}/\gamma \lesssim 1$, which is the case we expect to apply here due to silicon’s indirect bandgap, the lineshapes are independent of *ε* and we set *ε* = 0.1. This value should provide an upper bound for the internal quantum efficiency of our devices. The EQE we estimate is around 0.05%, but taking into account the reflection due to the dielectric constant mismatch at the silicon interface, an upper bound *ε* = 0.1 seems reasonable.

The simulations show an enhancement of the EL with magnetic field due to an increased number of states which overlap with the singlet ground state. This behavior arises from the fact that the spin–orbit interaction renormalises the effective hole *g*-factor (with effective Landé value *g*_h_/3 for total spin 3/2 holes), giving rise to a competition between the exchange interaction and the effective *g*-factor difference (and hence Zeeman energy) between electron and hole states. This leads to a mixing between singlet and triplet electron-hole spin configurations with a characteristic saturation field set by the competition between this Zeeman energy difference and the exchange term *B*_sat_~*J*/Δ*g*_eff_*μ*_B_. We find that the 300 K experimental lineshape can be reproduced with *J* = −0.75 meV, which provides an estimate for typical exchange interactions for transient bound states formed during electron-hole collisions at room temperature in silicon; as expected, *J* is smaller than the estimated exchange energy of 10 meV found for strongly bound excitonic states in Si nanocrystals^51^. Highlighting the importance of transient bound pairs, we show in the Supplementary Figure 3 that the positive MEL starts to decrease below 150 K, a temperature that matches the exciton binding energy (14.7 meV). This suggests that maximal sensitivity to magnetic field is achieved when the temperature is not too high, allowing interaction effects to show up, but not too small so that electron-hole encounter events do not result in irreversible binding. We emphasise that this model produces a MEL response, which does not depend on the direction of the external field, and can therefore explain the positive MEL component in our experiments (Fig. 4). Using our estimation for the exchange energy, we can check whether our assumption of weakly bound pairs is self-consistent. The typical size of the electron hole pair *d*_eh_ can be estimated by equating *J* with the pair Coulomb energy *e*^2^/4π*ε*_r_*ε*_0_*d*_eh_ (*ε*_r_ is the dielectric constant of Silicon), leading to *d*_eh_ approximately equal to 160 nm. For the pair to be well defined, the pair size has to be larger than the distance traveled by carriers due to thermal diffusion during the characteristic time-scale *ħ*/*J* of the interaction between the spins in the pair. With a thermal velocity *υ*_t_ = 10^5^ m s^−1^ for electrons at room temperature, the distance *ħυ*_t_*J*^−1^ is around 80 nm and is indeed smaller than pair size. Although our model reproduces the observed MEL lineshapes, it fails to correctly reproduce the magnitude of the effect. The simulated value in Fig. 5 for ΔEL/El at a magnetic field *B* of 7 T is around 0.1, as compared with the experimental 0.75. Theoretical MEL can increase up to 0.76 in the $\varepsilon \gg 1$ limit but this would imply a very high internal quantum efficiency, which we do not believe to hold in our devices. Instead, we suggest that multiple recombination attempts, and a more detailed description of carrier kinetics, as well as electron-valley mixing can further amplify the theoretical MEL magnitude. Such a complete theory is beyond the scope of this work.

In conclusion, we have reported a strong increase in the brightness of SiLEDs under a magnetic field. These LEDs were fabricated using a novel technique, which allowed us to simultaneously suppress classical MR effects and obtain effective emission. In analogy with magneto-optic models developed for organic semiconductors, we explained our results as arising from the difference in recombination rates between singlet and triplet electron-hole pairs, allowing the electron-hole pair exchange energy to be estimated from the experimental lineshapes. Our investigations suggest an optoelectronic approach to probe spin transport properties in silicon near room temperature, a material with promise for quantum information processing and spintronics. They also show that the control of spin properties can allow to substantially increase the brightness of SiLEDs, which can be important components for chip to chip optical communication.

## Methods

### Gas immersion laser doping

The out-of-equilibrium laser doping was performed in ultra high vacuum (10^−9^ mbar) with a XeCl 308 nm excimer laser of pulse duration 25 ns and energy 0.96 J cm^−2^. The precursor gas used for the B(P) doping is BCL_3_ (*PCL*_3_). The doping level is finely controlled by the number of laser shots, each shot introducing a fixed B dose corresponding to the surface density of chemisorbtion sites for the precursor gas on the Si surface. The vertical alignment of the *n* and *p* doped spots is performed by illuminating the substrate from the bottom and observing the sample with an infrared camera, thus visualizing the bottom spot when doping the top one.

### Magneto-electroluminescence

For magneto-electruminescence experiments the samples were mounted inside an optically accessible cryostat magnet providing a static magnetic field up to 7 T. The samples were thermalized at the measurement temperature through a Helium vapor. The EL was collimated outside the cryostat and focused on a commercial Silicon or Germanium photodetector a meter away from the cryostat. Taking into account the higher sensitivity of Germanium photodetectors at the silicon emission wavelength the same results were obtained with the two types of photodetectors.

### Data availability

The data and materials from this study are available on request from the corresponding author A.D.C.

## Electronic supplementary material

Supplementary Information
