# Supplementary material for: Room temperature magneto-optic effect in silicon light-emitting diodes
Source: Nat Commun. 2018 Jan 26;9:398. doi: 10.1038/s41467-017-02804-6 (PMC5785965; doi:10.1038/s41467-017-02804-6)
Supplement: Supplementary file 1 — Supplementary Information [file 41467_2017_2804_MOESM1_ESM.pdf]

Supplementary figure 1

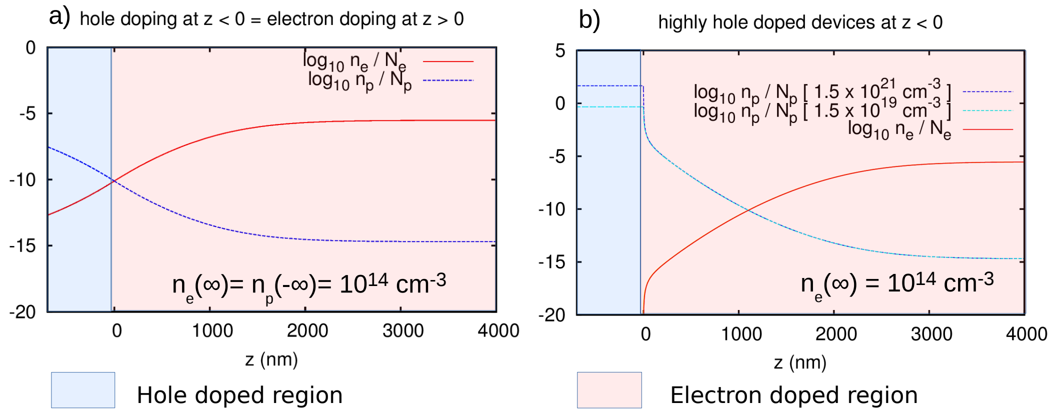

Supplementary Figure 1: **Theoretical charge density profiles in unbiased devices:** Theoretical dependence of the electron and hole density distributions (n_e and n_p respectively) at the p+/n interface for different doping levels in the $p+$ region as function of the vertical distance to the interface between the p+/n region (the densities are normalized by the effective density of states in the conduction/valence band defined in Eqs. (5,6)) from supplementary note 1 below. The left panel shows the density distribution for equal hole/electron doping in the p+/n regions . This case corresponds to the textbook case of a p/n junction with semiconducting range doping on both sides of the junction. As expected electron and hole densities curves cross in the depletion region of the diode which is centered at the interface at $z = 0$. For high hole doping in the p+ region (right panel) the deletion region is displaced into the n region to within a micron away from the p+/n interface. The electron/hole density profile in the electron doped region then depends only weakly on the doping on the p+ side (almost no change for hole doping densities between 1.5×10^{19} to 1.5×10^{21} cm^{-3}). On the other hand the amplitude of the potential barrier (see Figure 2 from main text) increases by 0.5 eV. This confirms our interpretation that the amplitude of the electron blocking potential is the relevant parameter to explain the increase in EL brightness in our experiments.

Supplementary Figure 2

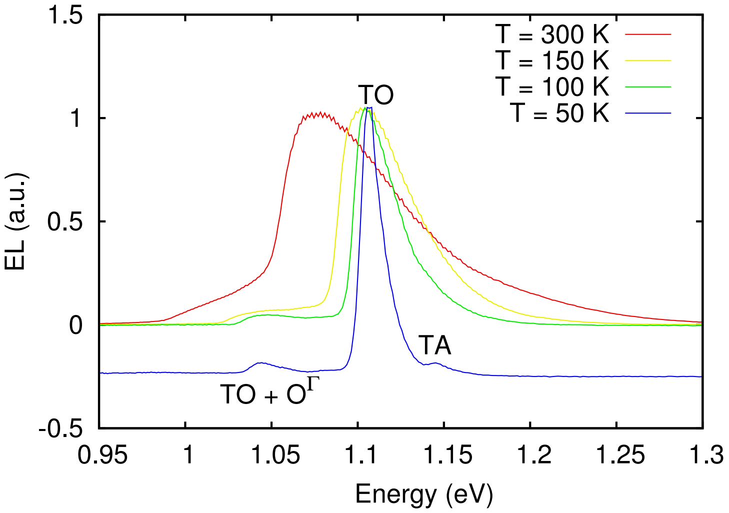

Supplementary Figure 2: **Low temperature electro-luminescence and EQE measurements:** Normalised electro-luminescence emission spectra from a lateral, $1.2 \times 10^{21}\mathrm{cm}^{-3}$ doping device at temperatures from 300 to 50 K at bias current $40mA$. The 50 K spectrum, shifted for clarity, reproduces the characteristic low temperature emission spectrum from silicon with well resolved $\mathrm{TO} + \mathrm{O}^{\Gamma}$, TO and TA phonon lines [2]. External quantum efficiency (EQE) was measured using a Thorlabs Ge photo-detector (similar results were obtained with a Si photo-detector and a Coherent Ge OP-2 IR detector of 1nW resolution) of known effective area and sensitivity mounted on a goniometer. The emitted power was measured for different inclination angles and as function of the distance between the photodetector and the SiLED confirming the expected scaling of the detected power as function of distance. The quantum efficiency was then deduced by summing contributions from different angles.

Supplementary Figure 3

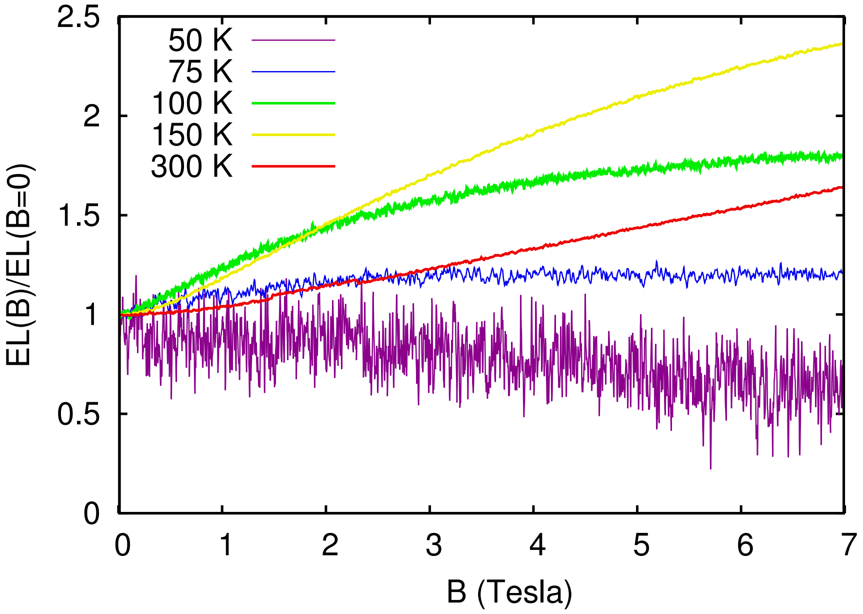

Supplementary Figure 3: **temperature dependence of the MEL effect:** Temperature dependence of MEL effect in a vertical device with a typical bias current of 20mA. Maximum MEL effect is observed around 150 K. Below this temperature the MEL starts to decrease with a weak residual negative MEL effect at 50 K that we attribute to magneto-diode effects. This characteristic temperatures of 150 K matches the binding energy of excitons in silicon (14.7 meV), suggesting that maximal sensitivity to magnetic field is achieved when the kinetic electron-hole energy is not too high to allow interaction effects to show up, but not to small so that electron-hole encounter events do not result in irreversible binding

.

Supplementary Figure 4

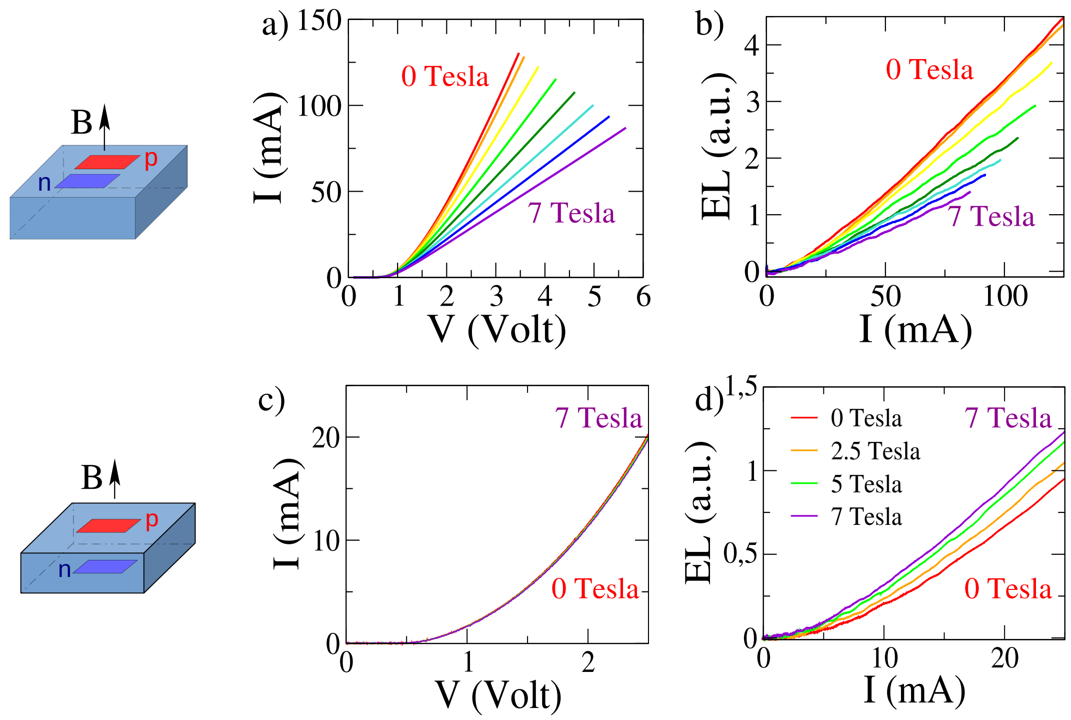

Supplementary Figure 4: **Comparison of MEL effect in lateral and vertical devices:** Panels a) and b) show the magnetic field effect at room temperature in a lateral devices with doping $1.2 \times 10^{21}\mathrm{cm}^{-3}$ where the magnetodiode effect dominates. a) Under magnetic field the resistance of the device increases as electron and hole trajectories are bent by the magnetic field. As the length of the electron-hole trajectory increases the carrier recombination probability is also enhanced, thus a smaller fraction of carriers reaches the interfaces where radiative recombination is efficient. This leads to a decrease of the EL with magnetic field at fixed current (see panel b). As shown in this figure the MEL is negative for the magnetodiode effect, with MEL and magnetoconductance effects having a similar magnitude.

Panels c) and d) show the magnetic field effect at room temperature in vertical devices for perpendicular magnetic field for a vertical device with $1.5 \times 10^{21}\mathrm{cm}^{-3}$ doping. As for the $3 \times 10^{21}\mathrm{cm}^{-3}$ doping device shown in the main text the magnetoresistance vanishes as the $I(V)$ curve is not changed by magnetic field (panel c) but a substantial increase in EL is observed in panel d) as opposed to the negative MEL in lateral geometry devices (panel b) .

Supplementary Figure 5

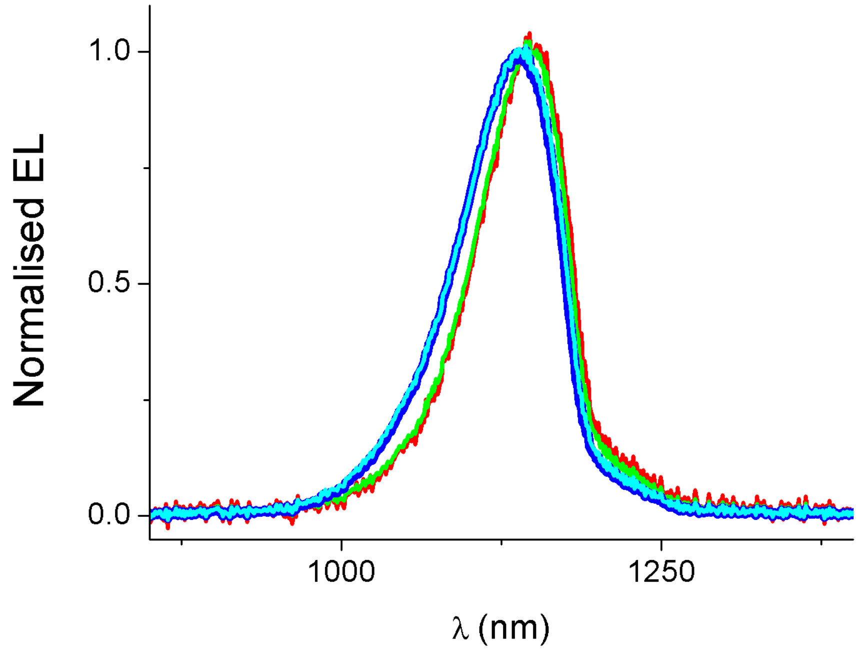

Supplementary Figure 5: **Doping dependence of the EL spectra at room temperature:** Spectra (normalized to maximum EL) of lateral (p+/n/n+) SiLED biased with 300 mA, for increasing concentration (red: $1.5\times10^{20}\text{cm}^{-3}$; green: $4.5\times10^{20}\text{cm}^{-3}$; blue: $1.5\times10^{21}\text{cm}^{-3}$; light blue: $4.5\times10^{21}\text{cm}^{-3}$. At room temperature the EL spectra are almost independent of the doping level. The small difference in line-shape is probably due to slightly different reflection conditions at highly doped interfaces for different doping levels.

Supplementary Figure 6

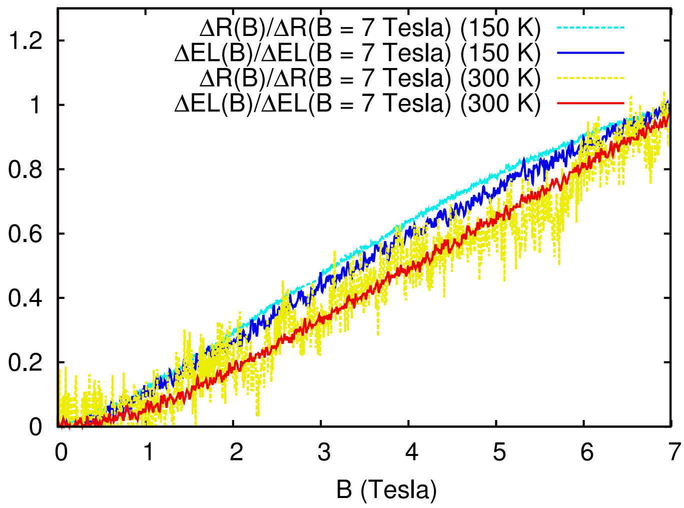

Supplementary Figure 6: **Rescaled data for Fig. 3:** Comparison between MEL and MR effects rescaled to their 7 Tesla values for the data shown on Figure 3 from the main text. A small increase in the rescaled resistance consistent with an increasing radiative recombination yield is observed. However the MR effect is much smaller with values at 7 Tesla of around 2.2% at 150K and 0.5% at 300K (for comparison MEL is then respectively 275% and 80%) and we thus prefer to remain cautious on its interpretation.

Supplementary note 1, theoretical charge density profiles in unbiased devices

To compute the charge distribution in unbiased devices, we solve the Laplace equation on the V the electrostatic potential, $\epsilon_0\epsilon_r$ the dielectric permittivity, q the charge of the electron:

$$\epsilon_r\epsilon_0\partial_x^2 V = -q[n_e - n_p - C(x)] \tag{1}$$

where n_e electron density, n_p hole density, $C(x)$ the doping profile.

The densities n, p can be determined from the position of the Fermi level F relative to the bottom of the conduction band E_c and to the top of the valence band E_v :

$$n_e = N_e F_{1/2}(\beta F - \beta E_c) \tag{2}$$

$$n_p = N_p F_{1/2}(\beta E_v - \beta F) \tag{3}$$

In the above equation we introduced the notations $\beta = (k_B T)^{-1}$ and $F_{1/2}$ is the Fermi-integral:

$$F_{1/2}(y) = \frac{2}{\sqrt{\pi}}\int_0^\infty \frac{\sqrt{t}dt}{1+e^{t-y}} \tag{4}$$

and N_e, N_P which are effective density of states in the conduction/valence bands. They are given by the following formulas [1]

$$N_e = 12\left(\frac{m_e^* k_B T}{2\pi\hbar^2}\right)^{3/2} \tag{5}$$

$$N_p = 2\left(\frac{m_p^* k_B T}{2\pi\hbar^2}\right)^{3/2} \tag{6}$$

which lead to $N_e = 2.8\times10^{19}$ cm^{-3} and $N_p = 1\times10^{19}$ cm^{-3} at room temperature.

Finally the electro-chemical potential μ remains constant across the bilayer :

$$\mu = F + qV \tag{7}$$

This leads to the following self-consistent equation on the electrostatic potential (we now count the potential from the electro-chemical potential μ, thus $F = -qV$):

$$\epsilon_r\epsilon_0\partial_x^2 V = -q[N_e F_{1/2}(-qV\beta - E_c\beta) - N_p(qV\beta + E_v\beta) - C(x)] \tag{8}$$

Boundary conditions on the electrostatic potential are given by:

$$N_e F_{1/2}(-qV(\pm\infty)\beta - E_c\beta) - N_p(qV(\pm\infty)\beta + E_v) = C(\pm\infty) \tag{9}$$

Choosing dimensionless units:

$$\partial_x^2 V = -F_{1/2}(-V - \beta\Delta/2) + N_r F_{1/2}(V - \beta\Delta/2) + C(x) \tag{10}$$

with $N_r = N_p/N_e$.

Here the potential is in units of temperature $k_B T = \beta^{-1}$, $C(x)$ in units of N_e and the length-scale λ is set by

$$\lambda = \sqrt{\frac{\epsilon_R\epsilon_0}{q\beta N_e}} \tag{11}$$

with room temperature values for silicon:

$$\beta^{-1} = 26 \text{ meV} \tag{12}$$

$$\lambda = 0.72 \text{ nm} \tag{13}$$

$$\beta\Delta = 43 \tag{14}$$

Thus we are thus finally lead to the equation:

$$\partial_x^2 V = -F_{1/2}(-V - \beta\Delta/2) + N_r F_{1/2}(V - \beta\Delta/2) - C_p\eta(-x) + C_e\eta(x) \tag{15}$$

$$= \partial_V\left[F_{3/2}(-V - \beta\Delta/2) + N_r F_{3/2}(V - \beta\Delta/2)\right] - C_p\eta(-x) + C_e\eta(x) \tag{16}$$

where all the coefficients are known ($F_{3/2}(x)$ is the complete Fermi-integral).

For $x > 0$, this can be integrated to:

$$H_e = \frac{(\partial_x V)^2}{2} - \left[F_{3/2}(-V - \beta\Delta/2) + N_r F_{3/2}(V - \beta\Delta/2) + C_e V\right] \tag{17}$$

and for $x < 0$

$$H_p = \frac{(\partial_x V)^2}{2} - \left[F_{3/2}(-V - \beta\Delta/2) + N_r F_{3/2}(V - \beta\Delta/2) - C_p V\right] \tag{18}$$

Combining the two conservation laws allows us to find the potential $V(0)$:

$$H_p - H_e = (C_e + C_p)V(0) \tag{19}$$

Starting from $V(0)$ the potential and density profiles can then be obtained by direct integration of the equations of motion.

For devices under bias the drift-diffusion equations were solved using a finite elements method.

Supplementary references :

[1] S.M. Sze, *Physics of semiconductor devices*, A Wiley, ISBN 0-471-09837-X
[2] Miin-Jang Chen, Eih-Zhe Liang, Shu-Wei Chang, and Ching-Fuh Lin a), Jour. of Appl. Phys. **90** 789 (2001)
